# Estimating a 3D Human Skeleton from a Single RGB Image by Fusing Predicted Depths from Multiple Virtual Viewpoints †

**DOI:** 10.3390/s24248017

**Published:** 2024-12-15

**Authors:** Wen-Nung Lie, Veasna Vann

**Affiliations:** Department of Electrical Engineering, Center for Innovative Research on Aging Society (CIRAS), Advanced Institute of Manufacturing with High-Tech Innovations (AIM-HI), National Chung Cheng University, Chia-Yi 621, Taiwan; vanveasna1997@gmail.com

**Keywords:** 3D human skeleton estimation, 3D human pose, deep learning, multi-view, virtual viewpoints

## Abstract

In computer vision, accurately estimating a 3D human skeleton from a single RGB image remains a challenging task. Inspired by the advantages of multi-view approaches, we propose a method of predicting enhanced 2D skeletons (specifically, predicting the joints’ relative depths) from multiple virtual viewpoints based on a single real-view image. By fusing these virtual-viewpoint skeletons, we can then estimate the final 3D human skeleton more accurately. Our network consists of two stages. The first stage is composed of a two-stream network: the Real-Net stream predicts 2D image coordinates and the relative depth for each joint from the real viewpoint, while the Virtual-Net stream estimates the relative depths in virtual viewpoints for the same joints. Our network’s second stage consists of a depth-denoising module, a cropped-to-original coordinate transform (COCT) module, and a fusion module. The goal of the fusion module is to fuse skeleton information from the real and virtual viewpoints so that it can undergo feature embedding, 2D-to-3D lifting, and regression to an accurate 3D skeleton. The experimental results demonstrate that our single-view method can achieve a performance of 45.7 mm on average per-joint position error, which is superior to that achieved in several other prior studies of the same kind and is comparable to that of other sequence-based methods that accept tens of consecutive frames as the input.

## 1. Introduction

In recent years, 3D human pose estimation has become a core component of several technologies in applications that use imaging sensors, such as virtual/augmented reality (VR/AR), action recognition [1], gesture recognition, video surveillance [2], care for the elderly [3], human–computer interaction (HCI), and human–robot collaboration (HRC) [4].

The kinds of sensor input for 3D human pose estimation can be diverse, such as a single monocular RGB image [5,6], an RGB image sequence [7,8], multi-view RGB images [9,10], and RGB-D images [11]. Multi-view RGB images, RGB image sequences, and RGB-D images are capable of providing explicit or implicit 3D geometrical information, whereas 3D human pose estimation from a single RGB image remains a difficult task due to the possible ambiguity in predicting depth from 2D information. To overcome this problem, some researchers proposed the use of a sequence of RGB images [7,8] as the input. By using image sequences, spatial and temporal variations can be exploited to explore the information of a 3D shape from motion and, thus, enhance the accuracy of the estimated 3D skeletons. However, a long sequence of input images will not only cause a time delay but also form a heavy burden in computations. On the other hand, an input of multi-view RGB images [9,10] is also capable of boosting the accuracy of 3D estimation through the principle of triangulation. One of the drawbacks is its requirement of several cameras from multiple viewpoints and a larger space in operation, which thus restricts its applications (e.g., in athletic training, health diagnosis, etc.). RGB-D 3D cameras relying on actively illuminated infrared signals are more expensive than traditional 2D cameras. Additionally, their depth measurement accuracy is still not reliable enough. Even though the three kinds of methods mentioned above have been proven to lead to significant improvements in achieving accurate 3D human pose estimation, their aforementioned deficiencies make the use of a single RGB image (which has lower system complexity and cost) as the input is an open issue that is deserving of exploration.

Estimating 3D human poses from a single RGB image presents significant challenges compared with multi-view-based and sequence-based methods. A primary issue is depth ambiguity, where a given 2D projection can correspond to different 3D structures, making accurate depth estimation difficult without additional perspectives. Occlusion handling is also problematic. Single-view systems struggle due to depth ambiguity and hidden body parts, unlike multi-view approaches, which benefit from 3D geometry and fewer occlusions, or sequence-based methods, which explore 3D shapes using temporal motion information. Due to the deficiencies above, it is more challenging to achieve high pose estimation accuracy for single-view methods.

Inspired by the performance of the multi-view approach and the simplicity of a single-image approach, in this study, we propose a new method that incorporates their respective advantages. Our system accepts a single RGB image as the input, predicts enhanced 2D skeletons using real viewpoints and multiple virtual viewpoints, and then combines them through a fusion module to predict the final 3D human skeleton.

Our contributions can be summarized as follows:

- We propose a 3D human skeleton estimation method that requires only a single monocular RGB image as the input, making our system realistic in many applications.
- We propose a two-stream method for predicting enhanced 2D skeletons with real and virtual viewpoints, and the outputs are then processed and fused via a cropped-to-original coordinate transform (COCT) module, a depth-denoising (DD) module, and a fusion module (FM) to regress the final 3D human skeletons.
- Our proposed method outperforms single-image-based methods when evaluated on the Human3.6M dataset [12] and achieves a performance comparable to that of state-of-the-art (SOTA) methods based on a long image sequence.

## 2. Related Work

In general, 3D human skeleton estimation methods can be categorized into “top-down” and “bottom-up” methods. Top-down methods require an object detector (e.g., the well-known YOLO [13]) as a pre-stage to localize an ROI (region of interest) from which the skeleton can be estimated. On the other hand, bottom-up methods (such as the OpenPose [14]) detect all individual joints in the full frame and then link proper ones to form complete or partial skeletons. Top-down methods are characterized by higher accuracy but suffer from a restricted capability for single-person skeleton detection at a time. Bottom-up methods have lower accuracy but are beneficial for multi-person skeleton detection and, thus, have a higher speed for the full frame. Our current method is essentially categorized as “top-down”. Among the top-down methods, we will review some prior works using two types of inputs that have been popular in recent years.

### 2.1. Single-View Methods

Wu et al. [15] proposed the prediction of several informative feature maps from a single RGB image input, such as 2D heatmaps, limb depth maps, and hidden feature maps, which are then concatenated together and fed into a 3D lifting module to regress the 3D human pose. To overcome the depth ambiguity and poor generalization due to overfitting on background features when lifting 2D joint locations to generate 3D poses, Zhou et al. [16] proposed a pose-guided transformer layer and an adaptive feature selection module that more effectively utilize image cues while mitigating overreliance on dataset-biased background information. Their pose-guided transformer layer was designed to selectively enhance the attention on relevant image features, reducing the focus on irrelevant background information. On the other hand, their adaptive feature selection module was designed to further refine the model’s focus, as it prunes less important image features based on their attention scores.

Many new architectures have been proposed to predict the 3D human pose from the predicted 2D human pose [17,18]. Kang et al. [17] proposed a grid convolution (GridConv) method to lift a 2D single-view pose to a 3D human pose. Their GridConv is built upon a revolutionary semantic grid transformation (SGT) that maps the irregular graph-structured human pose joint by joint onto a regular weave-like grid pose representation by using a binary assignment matrix. This allows layer-wise feature learning using GridConv operations. Xu et al. [18] proposed a novel architecture that enables hierarchical feature learning across multiple skeletal resolutions by using new pooling and up-pooling operations that allow the model to process human pose information across different scales, ensuring the effective capture of both local joint relationships and broader skeletal contexts.

Li et al. [19] addressed limitations in existing models, particularly issues with generalization on rare and complex poses due to small datasets. They proposed a CEE-Net to generate more diverse poses by expanding the distributions of joint angles beyond the training set, ensuring that the generated poses remain biomechanically realistic. On the other hand, a multi-layer perceptron (MLP)-based body-part grouping network that decouples the dependencies between joints was designed for pose estimation. By focusing on both within-group and between-group joint relationships, the estimator improves robustness to complex and unseen poses. Gong et al. [20] proposed a novel auto-augmentation framework (PoseAug) that learns to augment 2D–3D pose pairs in an online and end-to-end manner. They overcame poor generalization in existing 3D human pose estimators by using an augmenter that optimizes augmentation through three operations: joint-angle adjustment, body size scaling, and viewpoint changes. This process is guided by discriminators that ensure that the generated poses remain plausible.

Similarly to the above CEE-Net [19] and PoseAug [20] approaches, we proposed a depth denoising (DD) module that works as a data augmentation during the training process. Our DD module is a simple yet effective method compared with the state-of-the-art methods.

### 2.2. Multi-View Methods

Multi-view methods have also been proposed to overcome the issue of depth ambiguity in prediction. Bai et al. [21] proposed an end-to-end correlative channel-aware fusion (C2AF) network that aims to fuse temporal information and information from multiple views. First, the network extracts robust temporal information using a two-stream structured encoder for each view. The intra-view and inter-view correlation matrixes are then constructed and fed into a channel-aware learnable fusion network to explore the global correlative patterns. Iskakov et al. [10] introduced two innovative approaches by using learnable triangulation methods. The first one is “algebraic triangulation”, which is enhanced with learnable confidence weights for each camera view. By estimating the confidence of each view, the method prioritizes reliable views and minimizes the impact of noisy or occluded ones. Then, 2D human poses and the learned confidence weights are fed to the algebraic triangulation module to estimate the 3D joint positions. The second approach is “volumetric triangulation”, which creates a 3D volumetric representation of the scene by projecting 2D feature maps from multiple views into a 3D grid. The 3D volume is then processed by a 3D convolutional neural network (CNN) to produce 3D heatmaps for each joint. Finally, these 3D heatmaps are processed with a soft-argmax operation to obtain the 3D pose.

The accuracy of the predicted 3D human pose is often limited due to the occlusions or inaccuracies in 2D pose estimation. To overcome these challenges, Qiu et al. [9] proposed a cross-view fusion scheme that leverages heatmap information from multiple views, allowing the system to correct errors caused by occlusions and improve the detection of challenging joints, such as wrists and ankles. To recover 3D poses from multi-view 2D heatmaps, the authors presented the Recursive Pictorial Structure Model (RPSM) to refine the search space for each joint iteratively, gradually narrowing down the locations with higher precision. Kim et al. [22] proposed a self-supervision approach that leverages multi-view images without the need for camera calibration. They introduced a cross-view self-fusion module (CSM) to correct 2D pose errors caused by factors such as occlusions by fusing information from multiple views and aligning joint predictions across these views based on epipolar geometry. To solve the depth-scale ambiguity, they employed a lifting network that converts 2D poses into 3D poses by enforcing multi-view consistency. Additionally, they introduced a reprojection mechanism that projects the predicted 3D pose back to the 2D image space to ensure consistency between the 3D pose and the original 2D observations.

With the advancement of the graph convolutional network (GCN), Hua et al. [23] proposed a simple yet effective cross-view U-shaped graph convolutional network (CV-UGCN) for weakly supervised 3D human pose estimation. First, 2D key points are extracted and then lifted to a coarse 3D pose by using the triangulation method. Finally, the coarse 3D pose is refined to obtain the final 3D pose by using the proposed CV-UGCN network.

All of the above methods require multi-view images as the input, thus placing some restrictions on real applications. In our system, only a real camera is needed, while joint depths in several other virtual viewpoints are predicted and then fused to boost the accuracy of 3D pose estimation.

## 3. Proposed Method

**Figure 1:**
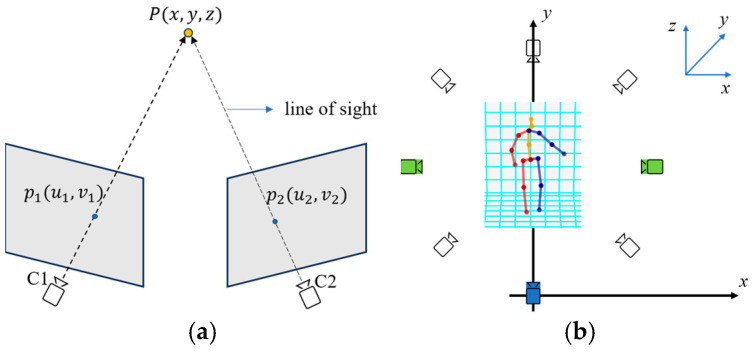

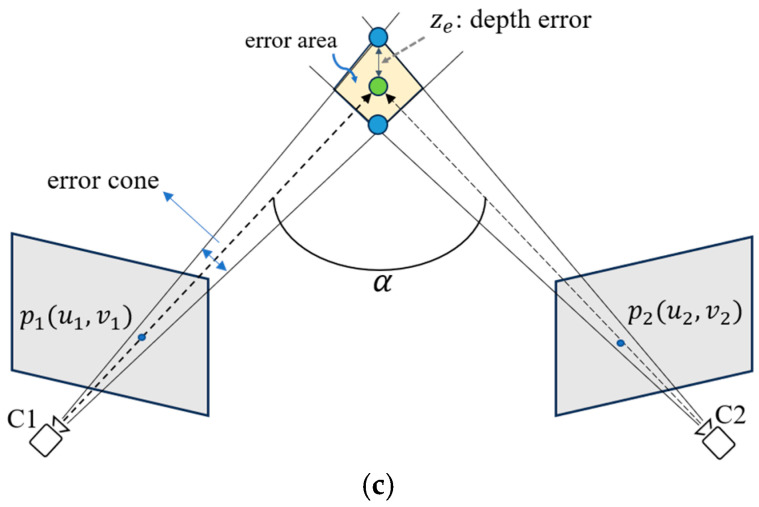
(**a**) Multi-view geometry; (**b**) our setup with multiple virtual viewpoints (the blue camera is real, the other *N* (here, *N* = 7) cameras are virtual, and the two green cameras are selected after experiments (Section 4.2.1)); (**c**) geometry for depth error analysis.

The ambiguity of depth prediction from a single RGB image remains a significant challenge in 3D human pose estimation, as monocular images inherently lack sufficient depth cues to accurately infer joint positions in 3D space. In many computer vision tasks, this depth ambiguity is resolved by using multiple cameras from different viewpoints, leveraging the triangulation principle or multi-view geometry (Figure 1a shows an example of two views) to estimate 3D information. However, the use of multiple real cameras in real-world applications (especially during the inference stage) presents several drawbacks, such as increased computational costs, hardware and space requirements, and system complexity (e.g., camera calibration is required).

**Figure 2:**
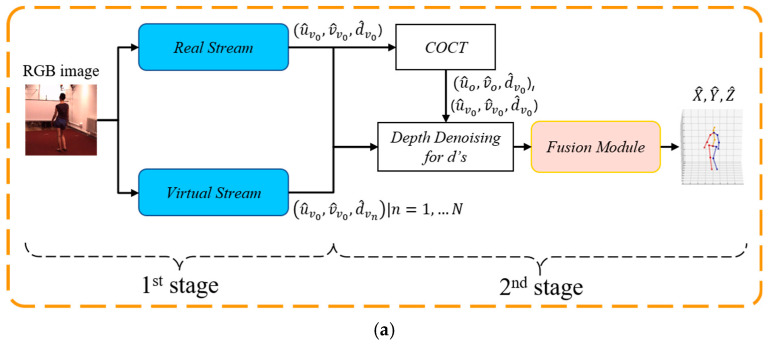

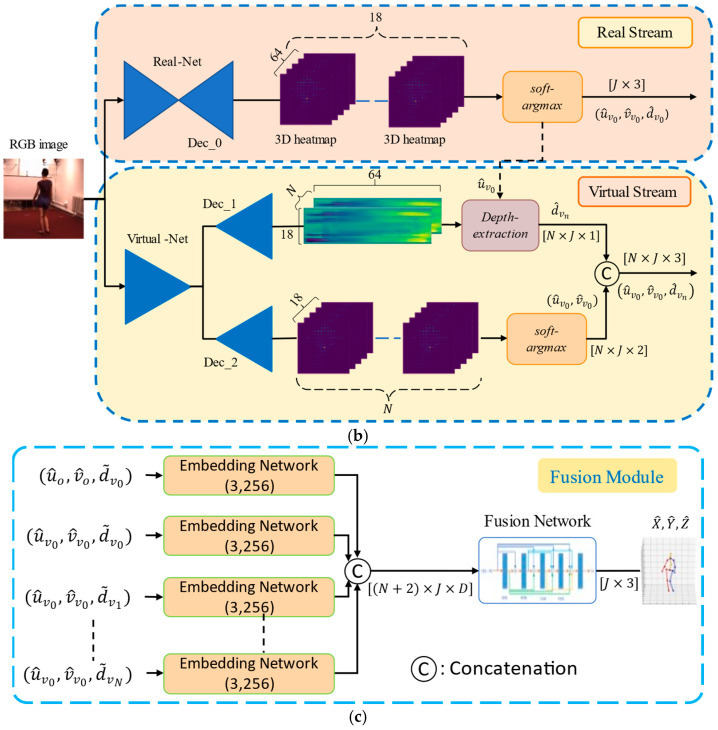
(**a**) Overall architecture of our proposed two-stream method. (**b**) Detailed architecture of the first-stage network, including the “real” stream (Real-Net) and virtual stream (Virtual-Net). (**c**) Detailed architecture of the fusion module (FM) in the second stage. *N* denotes the number of virtual viewpoints, *J* denotes the number of joints, and *D* denotes the dimension of the embeddings.

Figure 2 illustrates our proposed network architecture. In Figure 2a, the architecture is divided into two stages. The first stage is composed of a two-stream sub-network (real stream and virtual stream) that is designed to predict enhanced 2D skeletons with several viewpoints. As depicted in Figure 1b, *N* virtual cameras are arranged to encircle the target human by rotating around the *z*-axis. The “real” viewpoint (the blue one) represents the viewing direction corresponding to a real camera arranged in the system configuration, while the “virtual” viewpoints (the white ones) refer to the hypothesized ones without real cameras arranged therein. The second stage consists of several modules, including the cropped-to-original coordinate transform (COCT) module, depth denoising (DD) module, and fusion module (FM), to fuse the multiple enhanced 2D skeletons and lift them to form the final regressed 3D skeleton. The detailed design of each stage and module is given in the following subsections.

### 3.1. Stage 1: Real-Net and Virtual-Net

To overcome these limitations, we draw inspiration from the multi-view approach but aim to eliminate the need for multiple real cameras in realistic operation. Instead, we introduce virtual viewpoints to simulate the benefits of multi-view systems. By using virtual viewpoints, we can provide in-depth information from various perspectives, effectively addressing the depth ambiguity inherent in single-view geometry. In the first stage of our architecture, networks are designed to predict semi-3D human skeletons, which involve joints’ 2D image coordinates and relative depths from multiple real and virtual viewpoints. These predictions are then lifted to the full 3D space and integrated into the second-stage network to produce an accurate 3D human skeleton. This approach allows us to take advantage of multi-view geometry without the need for complex and resource-intensive setups with multiple real cameras. In this section, the detailed design of our Real-Net and Virtual-Net is provided.

Traditional two-stage 3D human pose estimation approaches often propose the prediction of pure 2D skeletons in the first stage by using HRNet [24], CPN [25], or other networks. However, we are motivated (based on our prior work [26]) to predict the “semi-3D skeleton” (or “enhanced 2D skeleton”, which includes 2D image coordinates and relative depth for each joint) rather than the traditional pure 2D skeletons. This semi-3D or enhanced 2D skeleton was also adopted in some other research [15] and might result in better accuracy once further converted/refined/lifted to a real 3D skeleton (i.e., with *x*, *y*, and *z* coordinates).

Considering the fact that there are no real cameras with virtual viewpoints (i.e., no real images are captured), our system predicts only the relative depth *d* (with respect to a “root” joint) of each joint in virtual viewpoints, but not their 2D image coordinates (*u*, *v*). These predicted relative depths ${\hat{d}}_{v_{n}}$ (*n*-th virtual view) are embedded in the 2D skeleton $\left({\hat{u}}_{v_{0}},\;{\hat{v}}_{v_{0}}\right)$ of the real camera $v_{0}$, thus forming *N* + 1 enhanced 2D skeletons $\left({\hat{u}}_{v_{0}},\;{\hat{v}}_{v_{0}},{\hat{d}}_{v_{n}}\right)$, *n* = 0, …, *N.* That is, the first-stage network predicts the real-view 2D skeleton $\left({\hat{u}}_{v_{0}},\;{\hat{v}}_{v_{0}}\right)$, as well as each joint’s relative depth ${\hat{d}}_{v_{n}}$ in all *N* + 1 viewpoints, as demonstrated in Figure 2a. The embedding of virtual-view depths into the real-view 2D skeleton has two advantages: lessening the prediction loading of the network and focusing on the ambiguity of the predicted depths.

The purpose of the Real-Net stream is to predict the enhanced 2D skeleton, which is denoted as $\left({\hat{u}}_{v_{0}}^{j},\;{\hat{v}}_{v_{0}}^{j},{\hat{d}}_{v_{0}}^{j}\right)$, *j* = 1, …, *J* (number of joints; here, *J* = 18). The input of the Real-Net is a single RGB image with a cropped size of 256×256 pixels. The output of the Real-Net is a number (18) of 3D heatmaps of size 64×64×64, where the spatial (in *u* and *v*) and the depth (*d*) dimensions are quantized into 64 levels. Each output element is transformed via the Softmax operator to reveal its probability of being the *j*-th joint. Via a Soft_argmax layer [27], it is easy to calculate the *J* predicted 2D joints with depths (*u*, *v*, *d*), forming an output of (J×3). Note that Soft_argmax is capable of transforming the heatmap to a regressed value, sharing the advantages of both heatmap (differentiable and easy convergence) and regression (end-to-end training with accessible output ground truths) approaches. The quantization into 64 levels in the (*u*, *v*, *d*) dimensions comes from considerations regarding speed and memory consumption.

The Virtual-Net stream, which differs from the Real-Net stream, is primarily designed to predict the relative depths of skeletal joints from multiple virtual viewpoints, which are denoted as ${\left\{{\hat{d}}_{v_{n}}\right\}}_{n=1}^{N}$. Additionally, it also predicts the 2D skeletal joint coordinates $\left({\hat{u}}_{v_{0}},\;{\hat{v}}_{v_{0}}\right)$ in the real viewpoint. As illustrated in Figure 2b, the virtual net stream consists of one encoder and two decoders, which process the same input image as the Real-Net.

The first decoder (Dec_1) generates *N* depth maps, each of size (64 × *J*), where *J* is the number of joints. Each depth map represents 3D tensors in terms of (depth, *u*-coordinate, and joint) and reveals the predicted depths for each joint at every possible *u*-coordinate. The relative depths ${\left\{{\hat{d}}_{v_{n}}^{j}\right\}}_{n=1}^{N}$ for each joint *j* are obtained by mapping these depth maps with the joint coordinate ${\hat{u}}_{v_{0}}$ predicted by the Real-Net.

The second decoder (Dec_2) generates (18 × *N*) heatmaps, each of size (64 × 64), representing the 2D joint locations in the real viewpoint. The final 2D skeletal joint coordinates $\left({\hat{u}}_{v_{0}},{\hat{v}}_{v_{0}}\right)$ are extracted using a soft-argmax operator that is applied to these heatmaps, similar to the process in the Real-Net.

The outputs from the Virtual-Net, i.e., the relative depths in virtual viewpoints ${\left\{{\hat{d}}_{v_{n}}^{j}\right\}}_{n=1}^{N}$ and 2D joint coordinates in the real viewpoint, $\left({\hat{u}}_{v_{0}},{\hat{v}}_{v_{0}}\right),$ are then combined to form $\left({\hat{u}}_{v_{0}},{\hat{v}}_{v_{0}},{\hat{d}}_{v_{n}}\right),\;n=1,\ldots ,N$. This combined output is subsequently fed into the second stage for further processing.

The 2D skeletal joints $\left({\hat{u}}_{v_{0}},{\hat{v}}_{v_{0}}\right)$ from the Real-Net and Virtual-Net are predicted based on the same ground truths and, thus, are expected to be approximate, while the ground truths of the relative depths in multiple virtual viewpoints are computed by geometrically rotating the real-view skeleton around the *z*-axis, as shown in Figure 1b. In this study, the network of ResNeXt-50 is chosen as the backbone of both the Real-Net and Virtual-Net.

### 3.2. Stage 2-1: Cropped-to-Original Coordinate Transform (COCT)

Accurate 3D human pose estimation requires the capture of both local and global image features. While local features provide detailed information about individual body parts, the global context is essential for resolving ambiguities among varying viewpoints. Traditionally, the input to a deep learning network is an ROI containing the target human, which is cropped from the original image and probably resized. Correspondingly, the 3D skeleton ground truth for training will remain the same, or the 2D skeleton ground truth will be transformed from the original coordinate system to the cropped image space. These cropping and resizing procedures will, however, cause the loss of global context information.

**Figure 3:**
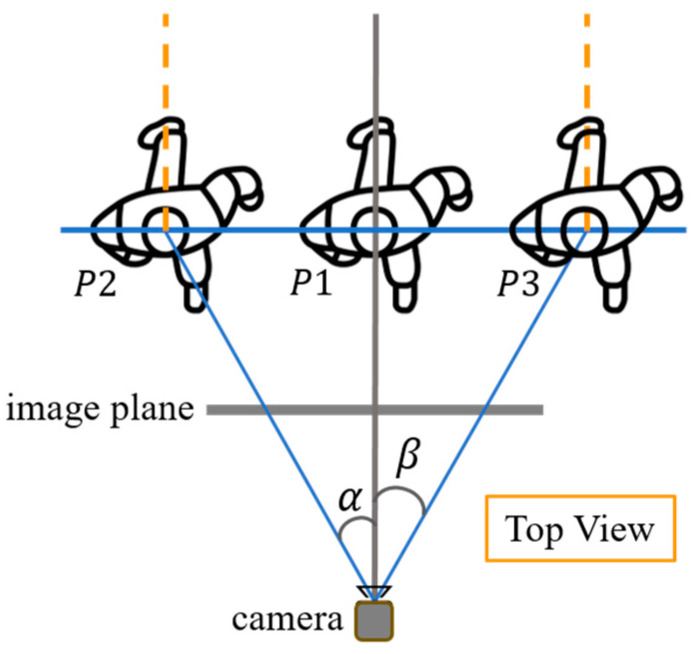
Global context information of humans (P1–P3) with the same 3D pose captured from different viewpoints (with horizontal viewing angles of −α, 0, and β, respectively) by the camera.

The scenario of Figure 3 shows three persons, P1–P3, with the same pose while standing at different positions in the camera coordinate space. Due to the characteristics of the camera perspectives, their 2D appearances exhibit some tiny differences (e.g., different self-occlusions or different horizontal projection lengths), hence resulting in different 2D skeletons. Nevertheless, their 3D poses, which are described by the relative 3D coordinates of each joint with respect to the selected root joint, are actually all the same. After image cropping for the ROI, the global context information (e.g., the location of the human: central, left, or right side) is lost and exists implicitly only in tiny perspective differences that might not be easily detected by the deep learning network.

To help the network learn global context information, a cropped-to-original coordinate transform (COCT) module is proposed in this study to provide explicit information on the original image coordinates. In both the training and inference phases, we transform the 2D skeletons from the cropped to the original image space by referring to the offset vector ${(o}_{u},o_{v}$) (in the original coordinate system) of the left-top corner of the ROI image based on the following equations:

(1) $${\hat{u}}_{o}=\frac{\left({\hat{u}}_{c}\times b_{w}+o_{u}\right)}{I_{w}}$$

(2) $${\hat{v}}_{o}=\frac{\left({\hat{v}}_{c}\times b_{h}+o_{v}\right)}{I_{h}}$$

where $\left({\hat{u}}_{c},{\hat{v}}_{c}\right)$ are the normalized coordinates (0~1.0) predicted after the network, $b_{w}\;and\;{\;b}_{h}$ are the width and height of the cropped ROI, $I_{w}\;and\;I_{h}$ are the width and height of the original image, and (${\hat{u}}_{o},{\hat{v}}_{o})$ are the normalized coordinates (0~1.0) in the original image space after the transformation.

This COCT-transformed skeleton of the real view is then concatenated with the predicted enhanced 2D skeleton in the cropped image space (see Figure 2c, the first and second branches) before being passed to the second-stage network. This dual representation (i.e., (${\hat{u}}_{o},{\hat{v}}_{o})$ and (${\hat{u}}_{v_{0}},{\hat{v}}_{v_{0}}$)) leverages the advantages of both the global (in the original image space) and the local (in the cropped image space) contexts, providing the following second-stage network with richer and more comprehensive input data.

### 3.3. Stage 2-2: Depth Denoising (DD) Module

It is normally ambiguous to predict depths from a single RGB image, and position errors mostly come from the depths of the estimated joints. In this study, a “depth denoising” technique is used to remove the noise of the joints predicted from the first-stage network. Because the 2D skeleton can be easily detected and has fewer errors, this denoising step is applied to the predicted depth component only. In denoising [28], Gaussian noise is added to the predicted depth output from the first-stage network, as expressed below:

(3) $${\tilde{d}}_{v_{n}}^{j}={\hat{d}}_{v_{n}}^{j}+N\left(\mu ,\;\sigma^{2}\right)$$

where $N\left(\mu ,\;\sigma^{2}\right)$ is the Gaussian noise with a mean μ and a variance $\sigma^{2}$, ${\hat{d}}_{v_{n}}^{j}$ is the predicted depth value for the *j*-th joint in viewpoint $v_{n}$, and ${\tilde{d}}_{v_{n}}^{j}$ is the output of the DD module. This DD module can also be considered as a data augmentation procedure since the noise is only applied in the training phase but is ignored in the inference phase. The procedure of using Gaussian noise to combat/remove the noise was also adopted in the design of a denoising autoencoder (DAE) [28].

### 3.4. Stage 2-3: Fusion Module (FM)

In the second stage, the fusion module (FM) is composed of parallel embedding networks that accept the depth-denoised enhanced 2D skeletons from all real and virtual viewpoints and a fusion network, as illustrated in Figure 2c. The embedding networks are used to transform the enhanced 2D skeletons to a high-dimensional feature space (from *J* × 3 to *J* × *D*, *D =* 256), and they are then concatenated and fed to the fusion network that follows. The embedding network can be implemented via an MLP (multiple-layer perceptron), which is a simple yet powerful feed-forward neural network that learns nonlinear relationships between the inputs and outputs. MLPs are often implemented as fully connected (FC) layers. The embedding networks can also be implemented in terms of a GCN (graph convolutional network) [29], which considers a human skeleton input as a graph with vertices (i.e., joints) connected via an adjacency matrix (specified with the standard connection of human skeletal joints via bones). GCNs capture the spatial relationships between connected joints, making them highly effective for modeling human poses and extracting their features. In the experimental section, we will provide a detailed ablation study on the performance of 3D skeleton estimation by using different kinds of embedding networks.

**Figure 4:**
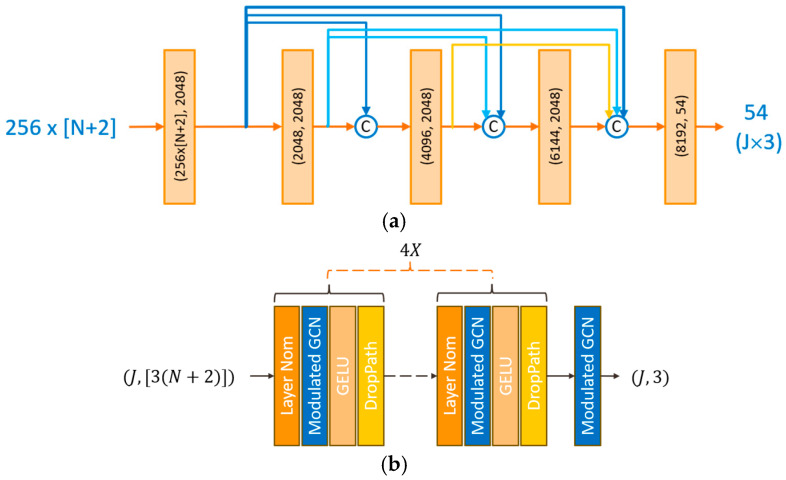
The architecture of the fusion network in the fusion module, where *N* is the total number of virtual viewpoints: (**a**) DenseFC network; (**b**) GCN.

As shown in Figure 2c, after the parallel embedding networks, the triplet outputs $\left(\hat{u},\hat{v},\tilde{d}\right)$ from all real and virtual viewpoint branches are concatenated together and flattened to form a 1D array (of dimension *J* × *D* × (*N* + 2)). In this way, the 2D spatial and depth information in the real and virtual viewpoints is combined. These concatenated data are then passed to a fusion network that follows for regression to a 3D human skeleton. Our fusion network is implemented in terms of densely and fully connected (DenseFC) layers, as proposed in the form of the DenseNet in [30]. Our fusion network architecture, DenseFC, is illustrated in Figure 4a, where the input is a set of *N* + 2 skeletal features (256 × (*N* + 2)), and the output provides the 3D skeleton (*J* × 3). Each block of this network is composed of a linear module followed by batch normalization, PReLU activation for nonlinear mapping, and a Dropout layer for regularization, but the last one contains only one linear module. These layers progressively refine the features, capturing intricate relationships between joints while mitigating overfitting. Dense connections ensure efficient feature reuse and mitigate vanishing gradient issues, facilitating deeper learning. This fusion network can also be designed by using a GCN [29], as shown in Figure 4b. By leveraging both the local joint information from the embedding network and the global spatial context from the fusion network, the FM is capable of combining depth information from multiple viewpoints. This approach reduces depth ambiguity and enhances the accuracy of 3D pose estimation, even from a single RGB image. To study the effects of different types of networks that can be used to design the fusion network, detailed ablation studies will be described in the experimental section.

### 3.5. Data Preprocessing and Virtual-Viewpoint Skeleton Generation

Both the input and output ground-truth data need to be well-prepared before training. Normalization, as a data preprocessing step, is used to transform the image and skeletal data into a common scale without distortions or information loss. For an image input, the popular z-score normalization is performed based on the mean *μ* and variance $\sigma^{2}$ for each of the R/G/B channels, where *μ* = (123.68, 116.28, 103.53) and *σ* = (58.40, 57.12, 57.38) are derived from the ImageNet dataset. For the enhanced 2D skeleton, the image coordinate (u, v) and the relative depth (d) of each joint need to be normalized to a common scale [0, 1.0] by dividing a normalization constant—256 pixels, 256 pixels, and 1000 mm, respectively—due to the different measurement units. Since (u, v,d) will be derived from the 3D heatmap (64 × 64 × 64) or the 3D depth map (64 × *J* × *N*) (as illustrated in Figure 2b) by applying a Soft-argmax or a lookup operation, the normalized (*u*, *v*, *d*) should be multiplied by 64 for the loss function calculation in training.

Similarly, we normalize the 3D skeleton ground truth in the second stage to the range of [−1, 1] by subtracting the root joint coordinates (here, “Pelvis” is selected as the root joint) and dividing the result by 1000 mm.

To obtain the ground truths of relative depths in virtual viewpoints, the *j*-th joint of the real-view 3D skeleton $S_{r}^{j}=\left(X_{r}^{j},Y_{r}^{j},Z_{r}^{j},1\right)\;(in\;homogeneous\;coordinates)$ can be transformed into the virtual viewpoint. The *j*-th joint of the 3D skeleton in the virtual viewpoint $v_{n}$ is denoted as $S_{v_{n}}^{j}=\left(X_{v_{n}}^{j},Y_{v_{n}}^{j},Z_{v_{n}}^{j},1\right)$, and $S_{v_{n}}^{j}$ can be obtained as follows:

(4) $$S_{v_{n}}^{j}=[R_{n}|T_{n}]S_{r}^{j}$$

where $R_{n}$ and $T_{n}$ represent the rotation and translation matrices for transformation between the real and the *n*-th virtual cameras. From {$S_{v_{n}}^{j}$}, *j* = 1, … *J*, the relative depth of joint j can be calculated. Note that $R_{n}$ and $T_{n}$ can be determined based on the viewpoint planning shown in Figure 1b.

### 3.6. Loss Functions

The loss function plays a pivotal role in guiding the learning process of a deep learning network, as it ensures that the predictions progressively align with the ground truths. Given the two-stage architecture of our method, we employ an independent multi-stage loss design. This approach ensures that each stage is optimized separately, allowing focused improvements in both the 2D + relative depth and the 3D pose estimation processes without the need to combine losses from different stages.

In the first stage, the training is further divided into two independent training processes: Real-Net and Virtual-Net. Real-Net is trained using the mean absolute error (MAE) between the predicted joints and their ground truths, as expressed below.

(5) $$L_{real\text{-}net}=\sum_{j=1}^{J}\left|{\hat{s}}^{j}-s^{j}\right|$$

where ${\hat{s}}^{j}=({\hat{u}}^{j},{\hat{v}}^{j},{\hat{d}}^{j})$ is the predicted enhanced 2D skeletal joint, $s^{j}=(u^{j},v^{j},d^{j})$ is the ground truth, and j is the joint index.

The loss function of the Virtual-Net is composed of both 2D and depth prediction losses.

(6) $$L_{virtual\text{-}net}={\alpha \cdot L}_{2D}+\beta \cdot L_{depth}$$

(7) $$L_{2D}=\sum_{n=1}^{N}\sum_{j=1}^{J}\left|{\hat{p}}_{v_{n}}^{j}-p_{v_{0}}^{j}\right|$$

(8) $$L_{depth}=\frac{1}{N.J}\sum_{n=1}^{N}\sum_{j=1}^{J}\left|{\hat{d}}_{v_{n}}^{j}-d_{v_{n}}^{j}\right|$$

where ${\hat{p}}_{v_{n}}^{j}=({\hat{u}}_{v_{n}}^{j},\;{\hat{v}}_{v_{n}}^{j})$ denotes the predicted 2D skeleton in the virtual viewpoint $v_{n}$, $p_{v_{0}}^{j}=(u_{v_{0}}^{j},\;v_{v_{0}}^{j})$ is the corresponding ground truth (inherited from the real viewpoint $v_{0}$), ${\hat{d}}_{v_{n}}^{j}$ denotes the predicted relative depth value, $d_{v_{n}}^{j}$ denotes the corresponding ground truth, *n* is the virtual viewpoint index, and α and β are the hyperparameters.

**Figure 5:**
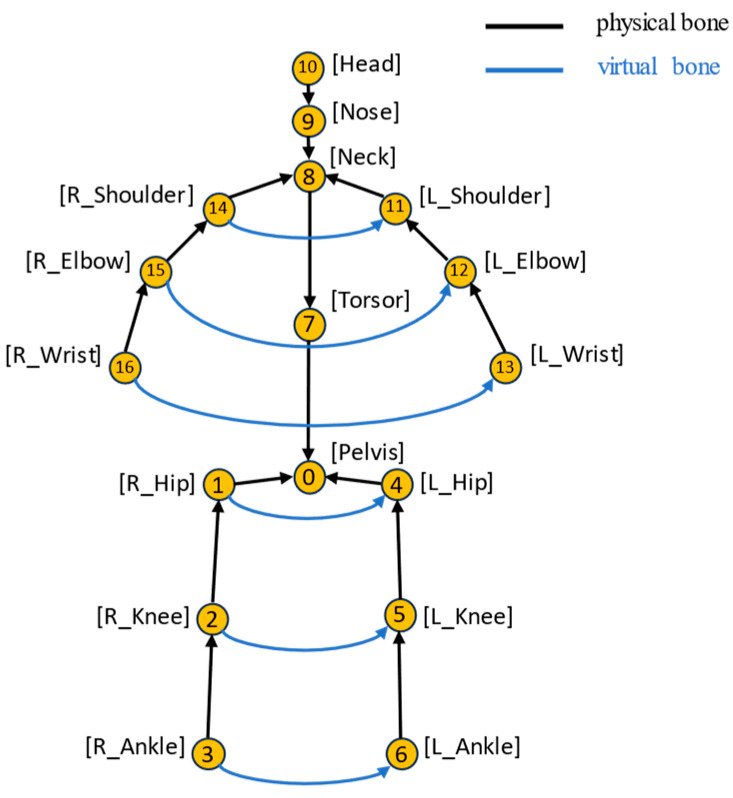
Illustration of the bone vector connections in our system.

The loss function of the second stage includes both joint and bone losses. The use of joint loss makes our network capable of learning the precise joint coordinates, while bone loss makes our network learn the skeleton’s spatial characteristics, which is helpful in enhancing the physical constraints between adjacent joints. To be consistent in the description of the joint loss, the bone loss is defined based on a bone vector pointing from a predefined starting joint to an ending joint. Figure 5 depicts all of the physical bones defined by Human3.6M (16 in total) and some virtual ones (6 in total) defined in our work. The virtual bones are connections between joints that are symmetric in the human skeleton, such as the left shoulder and right shoulder, left hip and right hip, etc. The total loss functions for the training of the second stage are defined as follows.

(9) $$L_{Fusion}\left(\hat{S},S\right)=\lambda_{bone}L_{bone}+\lambda_{joint}L_{joint}$$

(10) $$L_{joint}=\sum_{j=1}^{J}smoothL1({\hat{S}}^{j},S^{j})$$

(11) $$L_{bone}=\sum_{k=1}^{K}smoothL1({\hat{B}}^{k},B^{k})$$

(12) $$smoothL1\left(\hat{A},A\right)=\left\{\begin{array}{c} \frac{1}{2}({\left\|\hat{A}-A\right\|)}^{2}\;\;\;\;\;\;\;\;\;if\;\left\|\hat{A}-A\right\|<1\\ \left\|\hat{A}-A\right\|-\frac{1}{2},\;\;\;\;\;\;\;\;otherwise \end{array}\right.$$

where ${\hat{S}}^{j}$ and $S^{j}$ denote the prediction and the ground truth of the 3D coordinates for the joint j, respectively; ${\hat{B}}^{k}$ and $B^{k}$ denote the prediction and the ground truth of the *k*-th bone vector, respectively; and $\lambda_{bone}$ and $\lambda_{joint}$ are the hyperparameters for bone loss and joint loss, respectively. When calculating the losses, the *smoothL*1 metric, which is expressed in Equation (12), was used.

## 4. Experimental Results

### 4.1. Experimental Settings

In our system, virtual cameras were positioned around the *z*-axis at equal angular intervals to create 3D skeletons with virtual viewpoints, as shown in Figure 1b.

In this study, we used two popular datasets for network training: the Human3.6M [12] and MPII [31] datasets. The MPII dataset is a dataset for 2D human pose estimation. This dataset consists of 2D keypoint annotations for around 25k images extracted from online videos. The Human3.6M dataset is one of the most popular and largest datasets for 3D human pose estimation in indoor environments. It consists of 3.6 million human poses (2D/3D skeletal key points) and corresponding images captured by a high-speed motion capture system. Four synchronized high-resolution cameras running at 50 Hz were used to record the dataset, and they produced high-quality images of a variety of human postures and activities. This dataset contains activities by 11 professional actors in 17 scenarios. For the Human3.6M dataset, we followed the standard protocol by using 5 subjects (S1, S5, S6, S7, and S8) for training and 2 subjects (S9, S11) for testing. In the first stage, we used both Human3.6M and MPII as the training datasets, while in the second stage, only the Human3.6M dataset was used. The combination of the MPII and Human3.6M datasets in our first-stage training makes our model applicable in both indoor and more complex outdoor environments.

The performance of our proposed method is evaluated by using the standard evaluation metric used in previous studies. Protocol 1 is the mean per-joint position error (MPJPE) (Equation (13)), which computes the average of the Euclidean distance error in millimeters (mm) between the predicted skeleton and the ground truth. Protocol 2 is the Procrustes-aligned mean per-joint position error (PA-MPJPE), which represents the mean error between the prediction and ground truth after performing a rigid transformation via Procrustes Analysis.

(13) $$MPJPE=\frac{1}{F.J}\sum_{f=1}^{F}\sum_{j=1}^{J}||{\hat{S}}_{f}^{j}-S_{f}^{j}{\left|\right|}_{2}$$

where *f* is the frame index, ${\hat{S}}^{j}=({\hat{X}}^{j},{\hat{Y}}^{j},{\hat{Z}}^{j})$ is the *j*-th predicted 3D skeletal joint, $S^{j}=(X^{j},Y^{j},Z^{j})$ is the ground truth, J is the number of joints (*J* = 18), and F is the number of frames in testing.

We implemented our proposed method on the PyTorch platform on an Intel Core i9, with 64GB of RAM and an NVIDIA GeForce RTX 4090Ti. The Real-Net stream, with a computational cost of 8.71 GFLOPs (giga floating-point operations per second) per image in inference, required 15 h for training, while the Virtual-Net stream, at a computing cost of 9.42 GFLOPs, took 80 h for training. In the training of the first-stage model, the initial learning rate of 0.001 and the Adam optimizer were used. Real-Net was trained for 50 epochs, and Virtual-Net was trained for 100 epochs. The second-stage network (fusion module), with a computational cost of only 0.04 GFLOPs per image, required 3 h of training. The FM was trained for 250 epochs by using an initial learning rate of 0.001 with the AdamW optimizer. The hyperparameters for the loss functions were set as follows: α=1, $\beta =1,\lambda_{bone}$ = 10, and $\lambda_{joint}=1$.

### 4.2. Ablation Study

In order to evaluate the contribution of each component in our proposed method, we conducted ablation studies and experiments with diverse settings.

#### 4.2.1. Number of Virtual Viewpoints

First, the number *N* of virtual viewpoints is varied without utilizing depth denoising. Table 1 shows that all kinds of *N* > 0 are able to decrease the MPJPE, indicating that the accuracy of the 3D skeleton is improved via the fusion of the predicted relative depths from virtual viewpoints. When *N* equals 1, 2, 3, or 7, their performance is similar (MPJPE = 49.2 mm). Consequently, we decided to narrow down our focus to *N* = 1, 2, and 3 to conduct additional experiments concerning the arrangement of virtual cameras. Finally, it was observed that optimal results (MPJPE = 49.0 mm) could be obtained at *N* = 2, and the two virtual cameras are positioned at the clockwise 90° and the counterclockwise 90° intervals with respect to the real camera, as demonstrated by the green cameras in Figure 1b. This formed a three-viewpoint configuration of [real, v_left90, v_right90].

This result is reasonable when considering the analysis from Figure 1c. In Figure 1c, a depth error $z_{e}$ occurs from the interception of two error cones determined by pixels’ ($p_{1}\;and\;p_{2}$) positioning errors. The value of the depth error $z_{e}$ normally increases at a smaller angle α. For any two virtual viewpoints, the smallest positioning error (i.e., $min\_max(x_{e}$, $y_{e}$, $z_{e}$)) can be achieved when the angle *α* is equal to 90 degrees. This explains why the error performance of [real, v_left90, v_right90] is better than the symmetrical configuration [real, v_left120, v_right120]. Furthermore, increasing the number of virtual viewpoints beyond *N* = 2 did not result in significant improvements in accuracy because the angle *α* becomes smaller between any two adjacent virtual viewpoints. A larger *N* also increases the computational cost in inference. Our configuration of [real, v_left90, v_right90] offers an optimal trade-off, ensuring both precise 3D pose estimation and faster performance.

#### 4.2.2. Level of Depth Denoising

**Figure 6:**
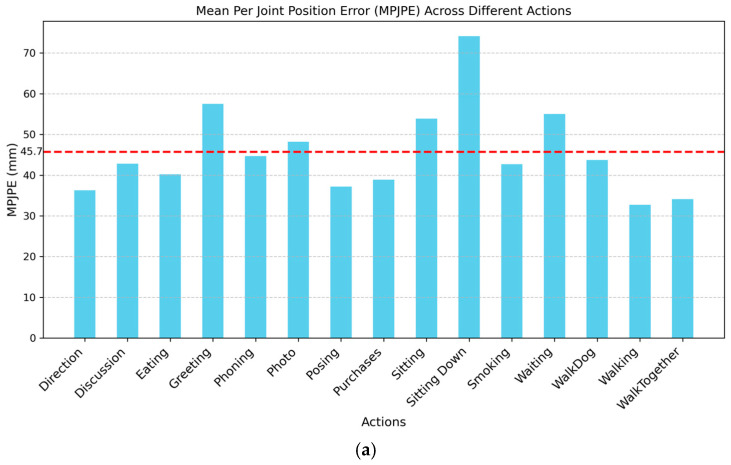

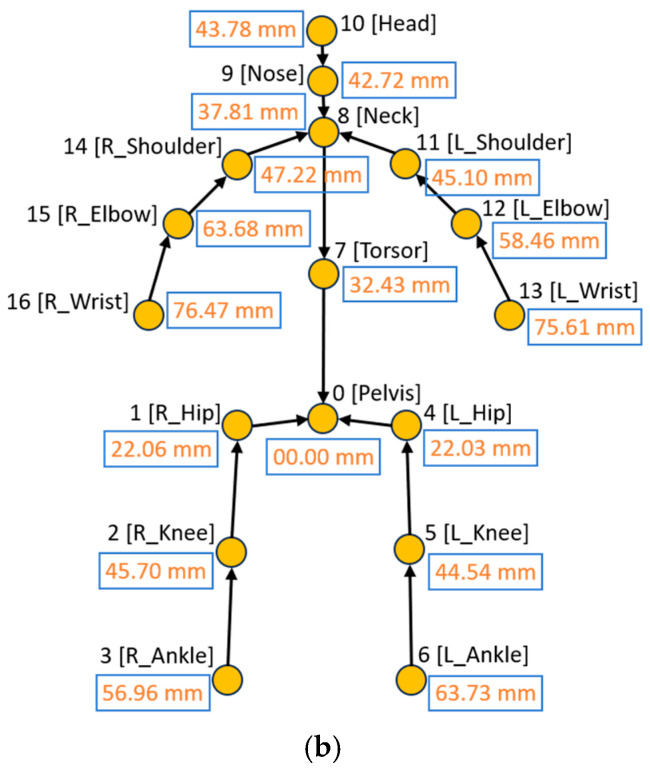
(**a**) Error distribution across different actions, where the dotted red line refers to the overall MPJPE value of 45.7 mm; (**b**) average MPJPE of each joint.

To assess the effectiveness of depth denoising, we opt for the optimal three-viewpoint configuration [real, v_left90, v_right90] and separately apply the depth denoising to the real viewpoint and virtual viewpoints. As depicted in Table 2, $\sigma_{r}$ and $\sigma_{v}$, representing the noise variances introduced to the joint depths predicted from the Real-Net and Virtual-Net, respectively, are varied. We adopted a strategy of individual optimization, i.e., varying $\sigma_{r}$ (at $\sigma_{v}$ = 0) and then varying $\sigma_{v}$ (at the optimized $\sigma_{r}$). It can be observed that $\sigma_{r}=0.022$ achieves the best average performance in the first round, and MPJPE and PA-MPJPE are further decreased to 47.48 mm and 36.97 mm, respectively, at $\sigma_{v}=0.01$. They correspond to Gaussian noises of σ = 10 mm (virtual) and 22 mm (real): 0.022 × 1000 mm = 22 mm and 0.01 × 1000 mm = 10 mm. These values are aligned with the joint position errors observed in our experiments (referring to Figure 6b), where errors mostly fall within 20~80 mm, ensuring that the noise levels reflecting the real-world depth variations are capable of improving the model’s generalization.

#### 4.2.3. Availability of the COCT Module

As aforementioned, the goal of the COCT module is to enhance the global context information. As illustrated in Figure 3, humans with the same 3D pose at different spatial locations should be predicted with similar performance (i.e., independent of the position). Here, we report the results when humans are present at different locations of the original image space to demonstrate the effectiveness of our COCT module.

First, the testing set is divided into three categories based on the distances of the human’s root joint to the frame center: small (at a distance of 0~99 pixels), medium (at a distance of 100~249 pixels), and large (at a distance of 250~375 pixels). As shown in Table 3, the performance in all three categories is improved with the availability of the COCT module. Specifically, the improvements in the “large” category (i.e., the humans are near the image boundary) are larger than in the “small” and “medium” categories. The effectiveness and impact of our COCT module in compensating for the human positions in the space are, thus, clear. It is noticeable that the main actions/poses for the three categories are the following: (1) “small”: sitting, sitting down, photo; (2) “medium”: greeting, walking together, phoning; (3) “large”: walk, walking a dog, walking together. Sitting, sitting down, and phoning are much more complex and have more self-occlusions, thus leading to higher estimation errors.

#### 4.2.4. Embedding Network and Fusion Network

To study the effectiveness and model size of different embedding networks and fusion networks (i.e., the fusion module) in our proposed method, they were designed based on three different backbones, including an MLP, DenseFC, and GCN. In this study, we followed [29] to design the GCN backbone in our network.

As shown in Table 4, it is observed that when the embedding network and the fusion network are designed using an MLP and the DenseFC network, respectively, the best MPJPE performance can be achieved at the expense of a larger FM size. Specifically, despite only leading to a moderate MPJPE performance, with the use of GCNs in both the embedding and fusion sub-networks, the model size is substantially smaller (1.5 MB or 0.25% compared with MLP + DenseFC), proving the efficiency of considering a human skeleton as a graph. In summary, MLP + DenseFC can be chosen when considering accuracy, GCN + GCN is the best in considering cost and efficiency, and GCN + DenseFC results in a tradeoff between accuracy and cost.

#### 4.2.5. Impact of Each Component in Our Proposed Method

Table 5 presents an analysis of the impact of each module in our proposed approach. As shown in Table 5, using only the real viewpoint data leads to the highest errors (MPJPE = 50.6 mm, PA-MPJPE = 38.1 mm). Incorporating *N* = 2 virtual viewpoints reduces these errors (MPJPE = 49.0 mm, PA-MPJPE = 37.5 mm), demonstrating the advantages of virtual viewpoints. Adding depth-denoising further decreases the MPJPE to 47.5 mm, while PA-MPJPE remains the same. When the COCT module is introduced, both MPJPE and PA-MPJPE are dropped further to 46.6 mm and 36.9 mm, respectively, indicating the importance of providing global context information via the original image coordinates. Finally, the embedding network (MLP) enables multi-view feature fusion instead of raw data fusion to further boost the performance to MPJPE = 45.7 mm and PA-MPJPE = 36.6 mm. Note that, in all cases, the fusion network adopting DenseFC is required to fuse and lift the multi-view enhanced 2D skeletons to a 3D skeleton.

### 4.3. Performance Comparison with State-of-the-Art (SOTA) Methods

We compare our proposed method with SOTA methods (all in the top-down category) based on training and evaluation of the Human3.6M dataset. All of the SOTA methods used for comparison are categorized as top-down methods. That is, an object detector (e.g., the well-known YOLO [13] or CenterNet [32]) is needed to crop the ROI for 3D skeleton estimation. Each time, only one ROI image of a single person is processed.

In Table 6, the parameter *T* represents the number of consecutive frames utilized as the input for 3D human skeleton estimation. A *T* value larger than 1 will result in a sequence-to-sequence conversion (i.e., *T* frames as input and *T* skeletons as output). However, only the central skeleton will be used for performance evaluation. Processing the sequence input is expected to require more intensive computation and a larger model. Our proposed method, which targets a single-frame input, demonstrates superior performance, with 45.7 mm in MPJPE and 36.6 mm in PA-MPJPE, outperforming the SOTA methods that similarly adopt *T* = 1 as a parameter (e.g., [16,19,29,33,34]). In [33] (HTNet), although they have a 3D pose refinement stage, our method performs better than theirs by 1.9 mm in MPJPE. Furthermore, our method also outperforms that of [19] (CEE-Net), which proposed the generation of diverse poses that work as a form of data augmentation (in contrast, our depth denoising module is simple and effective) by 1.6 mm.

In comparison with sequence-based (e.g., *T* = 31~351) methods, it is found that our results are comparable to theirs and even outperform some of them (e.g., [8,33,36,37,40]). In practical terms, using a long sequence of frames as input presents some drawbacks, such as increased network size, higher memory requirements, more intensive computation, and longer response times. In contrast, our approach, which is based on a single frame as an input, not only simplifies the system hardware/software but also leads to a quicker response time, making it a more cost-effective design.

### 4.4. Error and Cost Analysis

Figure 6a shows the error histogram across different actions or postures, where the dotted red line refers to the overall MPJPE value of 45.7 mm (Table 6). As shown in Figure 6a, simpler actions such as “walking” and “walking together” exhibit relatively low MPJPEs (at values of 32.7 mm and 34.1 mm, respectively). This suggests that our model can effectively handle actions with consistent and repetitive movements and minimal occlusions, where depth estimation is more straightforward.

In contrast, more complex actions such as “sitting down” and “greeting” show higher MPJPE values (74.1 mm and 57.5 mm, respectively). These actions often involve significant self-occlusions or challenging poses, such as crossing limbs or sudden changes in depth, which increase the difficulty of accurate 3D pose estimation from a single RGB image. Interestingly, the “phoning” and “photo” actions, which involve static gestures, show moderate error levels (44.7 mm and 48.2 mm, respectively). This could be attributed to the limited visibility of certain joints (e.g., hands near the face) and less distinctive depth cues. Future work could focus on boosting the model’s performance by incorporating additional training data with diverse occlusions or leveraging temporal sequence information to enhance robustness and filter the estimation noises.

The average MPJPEs for each individual joint are illustrated in Figure 6b. Notably, dynamic joints, such as the hands (wrists, elbows, and shoulders) and legs (knees and ankles), exhibit larger errors (MPJPE = 44.54 mm~76.47 mm) than more static joints, such as the torso, neck, and hips (MPJPE = 22.03 mm~37.81 mm). These results highlight the challenges in 3D pose estimation for dynamic body parts and suggest that additional strategies, such as temporal input or weighted loss, may be beneficial for improving the accuracy in these areas.

Regarding the resource and computational costs required in our network design, Real-Net, Virtual-Net, and FM (as shown in Figure 2) have model sizes of 405.9 MB, 528.1 MB (*N* = 2), and 590.6 MB (MLP + DenseFC), respectively. The corresponding computations required in terms of GFLOPs for each image in inference with Real-Net, Virtual-Net, and FM are 8.71, 9.42, and 0.04 GFLOPs (with only 0.0029 GFLOPs for the GCN + GCN implementation), respectively. Other SOTA methods, such as the popular CPN [25] and HRNet [24], require model sizes of 531 MB and 243.2 MB, respectively, to extract a 2D human skeleton. The model size for lifting a 2D skeleton to a 3D skeleton is, however, seldom reported. Table 7 illustrates a comparison with some selected SOTA methods.

As shown in Table 7, the adoption of virtual viewpoints introduces additional computational cost (9.42 GFLOPs) and network complexity (528.1 MB), as it requires the prediction of joints’ relative depths from multiple virtual viewpoints. However, this increased cost significantly improves the accuracy by resolving depth ambiguity and enhancing 3D pose estimation, as demonstrated by the reduction in the MPJPE from 50.6 mm to 45.7 mm (see Table 5). These trade-offs are justified by the performance gains observed in our experiments.

### 4.5. Visualized Results and Real Tests

**Figure 7:**
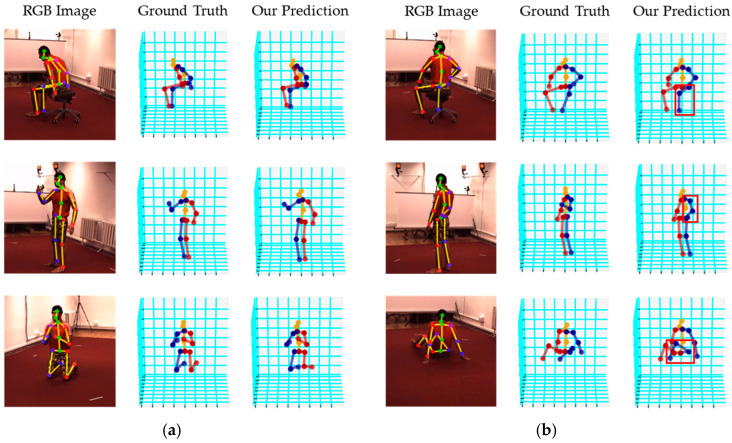
Visualized results on the Human3.6M dataset: (**a**) successful predictions; (**b**) failed predictions on some joints.

Figure 7 demonstrates the qualitative and visualized results of our proposed method on the Human3.6M dataset. The inference time for a 256 × 256 ROI image input is 0.0226 s per frame (equal to 44 FPS (frames per second)). As shown in Figure 7a, our proposed method could produce plausible 3D human skeletons similar to the ground truths with different action types. In complex situations such as heavy self-occlusions by humans themselves and intricate postures, our approach failed to predict certain joints correctly. For example, in Figure 7b, a leg is fully occluded by the chair stand (top), the right hand is fully occluded by the body (middle), and a leg is fully occluded by the body (bottom). In these challenging cases, our system actually predicts the occluded joints with a posture similar to the un-occluded one (e.g., the left hand was observed, but the right hand was occluded).

**Figure 8:**
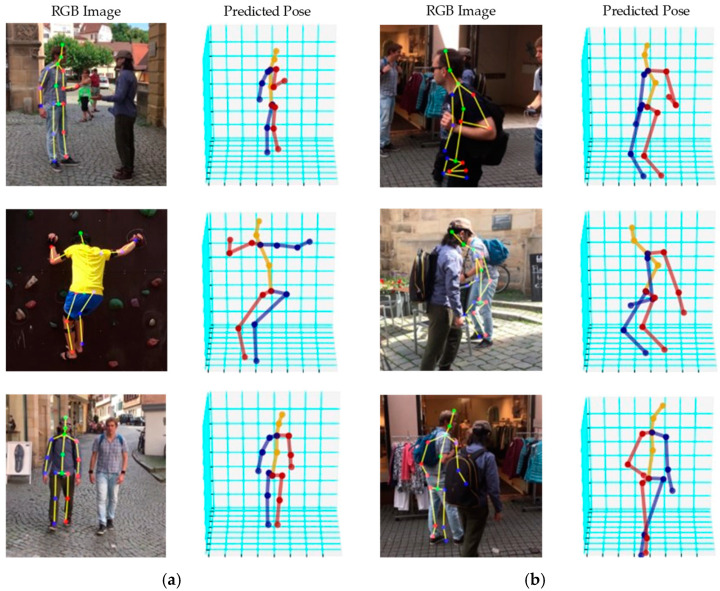
Qualitative results of the in-the-wild scenarios: (**a**) successful cases; (**b**) failed cases.

To demonstrate the generalization of our method, we provide some qualitative results for in-the-wild images captured using traditional camera sensors as a real test. Figure 8 shows some example images. For the real test, an object detector (such as the well-known YOLO [13] or CenterNet [32]) was used as a pre-stage to localize the human ROI first. Our algorithm then processed the ROI to predict the final 3D skeleton. The processing time (including that for the object detector) for this real application was, thus, 0.0535 s (about 19 FPS) for each frame and each person. As presented in Figure 8a, our proposed method could predict plausible 3D skeletons for different in-the-wild scenarios and complex backgrounds, even though our system was not trained with this kind of data. In challenging scenarios illustrated in Figure 8b, our technique failed to predict a reasonable skeleton. The difficult cases mostly came from situations in which two persons overlapped, and one was heavily occluded by the other, or an incomplete human was inputted (e.g., missing or truncated legs). Note that multiple ROIs can be detected and then processed one by one for multi-person skeleton estimation. Here, for simplicity and clarity, only the result of one human with the corresponding skeleton is shown.

## 5. Discussions and Conclusions

In this study, we introduced a novel approach for estimating 3D human skeletons from a single RGB image by fusing predicted depths from one real and multiple virtual camera viewpoints. Our proposed method uses a tradeoff between accurate multi-view and simple single-view methods by leveraging skeleton predictions with multiple virtual viewpoints. The two-stage architecture of our model, which includes Real-Net and Virtual-Net streams followed by a fusion module, is proven to be effective in predicting accurate 3D skeletons.

Our system, which was validated on the well-known Human3.6M dataset, demonstrates MPJPE = 45.7 mm and PA-MPJPE = 36.6 mm, thus outperforming SOTA methods that similarly accept *T* = 1 frame as the input, and it presents competitive performance in comparison with sequence-based SOTA methods that have *T* >> 1. Our design is more practical considering its monocular and single-frame configurations.

The efficiency and performance of our model make it highly suitable for a range of real-world applications. In healthcare monitoring or diagnosis (e.g., Parkinson’s or Alzheimer’s disease), the ability to accurately estimate 3D poses from a single camera enables non-intrusive tracking of patients’ movements or actions and support in rehabilitation and physical therapy. In virtual and augmented reality (VR/AR), our method can improve user interactions by providing precise pose tracking without requiring complex multi-camera setups. Additionally, the model can be applied to human–robot interaction, where real-time 3D pose estimation is essential for effective communication and collaboration between humans and robots.

Despite the success of our model, some limitations remain, particularly in scenarios where heavy self-occlusions or complex interactions between multiple humans are present. We may resort to the use of “bottom-up” methods, e.g., the popular OpenPose tool [14] or [41], which detect individual joints in the full frame and then link proper ones to form complete or partial skeletons. Unfortunately, bottom-up methods are seldom developed for 3D skeleton estimation and have less accuracy. One of the possible solutions to overcome the aforementioned deficiencies might be preprocessing with an instance segmentation technique to remove disturbing persons or objects before the 3D skeleton estimation starts.

Further improvements will be focused on the limitations identified in this work. One key direction is predicting the complete virtual-view skeleton $\left({\hat{u}}_{v_{n}},{\hat{v}}_{v_{n}},{\hat{d}}_{v_{n}}\right)$ but not just the relative depths of the joints embedded in the real-view 2D skeleton, i.e., $\left({\hat{u}}_{v_{0}},{\hat{v}}_{v_{0}},{\hat{d}}_{v_{n}}\right)$, as addressed in Figure 2b,c. This is intended to create a more cohesive and accurate virtual-view representation, hence improving 3D pose consistency across different viewpoints. To further address the issue of heavy occlusions, top-down methods capable of predicting incomplete 3D human poses are challenging, as they cannot compromise the accuracy of the final 3D pose even when significant occlusions occur. Finally, integrating short-term (e.g., less than 10 frames) temporal information still remains a priority for more accurate prediction.

## Figures and Tables

**Table 1:** Comparison of different numbers of virtual viewpoints. This result was obtained without depth denoising. The last row (v_left90, v_right90) denotes a three-viewpoint configuration that includes the real viewpoint, virtual viewpoint at the left 90°, and virtual viewpoint at the right 90°.

Number of Virtual Viewpoints (*N*)	MPJPE (mm)	PA-MPJPE (mm)
0 (real viewpoint only)	50.6	38.1
1	49.2	37.5
2	49.2	37.6
3	49.2	37.7
4	49.3	37.8
5	49.3	37.6
6	49.3	37.6
7	49.2	37.7
8	50.0	38.3
9	49.3	37.7
10	49.3	37.7
2 (v_left90, v_right90)	49.0	37.5

**Table 2:** Comparison of different variances for depth denoising with the three-viewpoint configuration [real, v_left90, v_rught90]$.\;\sigma_{r}$ and $\sigma_{v}$ are noise variances for predicted depths in the real and virtual viewpoints, respectively. The bolded/underlined numbers represent the best performance in each group of experiments.

$\sigma_{r}$	$\sigma_{v}$	MPJPE (mm)	PA-MPJPE (mm)
0 (none)	0 (none)	49.00	37.5
0.010	0	47.90	**37.0**
0.02	0	47.57	37.1
0.022	0	47.52	37.2
0.025	0	47.61	37.4
0.030	0	47.93	37.6
0.040	0	48.81	38.7
0.022	0.001	47.60	37.2
0.022	0.005	47.50	**37.0**
0.022	0.010	**47.48**	37.5
0.022	0.015	47.78	37.3
0.022	0.020	47.81	37.1

**Table 3:** Comparison of improvements in MPJPE with different locations of the humans when considering their distances in the image from the root joint to the frame center.

Human’s Position	Distance (pixel)	MPJPE (mm) (w/o COCT)	MPJPE (mm) (with COCT)	Improvement (mm)
Small (34.17%%)	0–99	51.01	50.14	0.87
Medium (61.74%)	100–249	45.82	44.95	0.87
Large (4.09%)	250–375	43.03	41.57	1.46

**Table 4:** Comparison of different embedding and fusion sub-network designs.

Embedding Network	Fusion Network	MPJPE (mm)	PA-MPJPE (mm)	Model Size (MB)
MLP	DenseFC	45.78	36.61	590.6
MLP	MLP	46.82	37.07	360.0
MLP	GCN	47.60	37.87	219.9
GCN	DenseFC	46.12	36.61	313.1
GCN	MLP	46.77	37.23	309.4
GCN	GCN	46.43	37.18	1.5

**Table 5:** Impact on the MPJPE for each module in our proposed approach.

Real Viewpoint	Virtual Viewpoints	Depth Denoising	COCT	Embedding Network	Fusion Network	MPJPE (mm)	PA-MPJPE (mm)
**✓**					**✓**	50.6	38.1
**✓**	**✓**				**✓**	49.0	37.5
**✓**	**✓**	**✓**			**✓**	47.5	37.5
**✓**	**✓**	**✓**	**✓**		**✓**	46.6	36.9
**✓**	**✓**	**✓**	**✓**	**✓**	**✓**	45.7	36.6

**Table 6:** Comparison of our results with other SOTA methods under Protocol #1 (top) and Protocol #2 (bottom) (*T*: number of input frames used for estimation; Bold: the best one).

**Protocol #1**	**Dir.**	**Disc.**	**Eat**	**Greet**	**Phone**	**Photo**	**Pose**	**Purch.**	**Sit**	**SitD.**	**Smoke**	**Wait**	**WalkD**	**Walk**	**WalkT**	**Avg.**
CEE-Net [19] (*T* = 1)	-	-	-	-	-	-	-	-	-	-	-	-	-	-	-	47.3
Zou et al. [29] (*T* = 1)	45.4	49.2	45.7	49.4	50.4	58.2	47.9	46.0	57.5	63.0	49.7	46.6	52.2	38.9	40.8	49.4
Lifting by Image [16] (*T* = 1)	44.9	46.4	42.4	44.9	48.7	40.1	44.3	55.0	58.9	47.1	48.2	42.6	36.9	48.8	40.1	46.4
LCMDN [34] (*T* = 1)	42.0	47.1	44.5	48.2	54.5	58.1	44.0	45.8	57.9	71.4	52.0	48.7	52.7	41.3	42.3	50.0
HTNet [33] (*T* = 1)	-	-	-	-	-	-	-	-	-	-	-	-	-	-	-	47.6
HTNet [33] (*T* = 27)	-	-	-	-	-	-	-	-	-	-	-	-	-	-	-	46.1
MHFormer [35] (*T* = 351)	39.2	43.1	40.1	40.9	44.9	51.2	40.6	41.3	53.5	60.3	43.7	41.1	43.8	29.8	30.6	43.0
PoseFormer [36] (*T* = 81)	41.5	44.8	39.8	42.5	46.5	51.6	42.1	42.0	53.3	60.7	45.5	43.3	46.1	31.8	32.2	44.3
PoseFormer [36] (*T* = 27)	-	-	-	-	-	-	-	-	-	-	-	-	-	-	-	47.0
Chen et al. [37] (*T* = 243)	41.4	43.5	40.1	42.9	46.6	51.9	41.7	42.3	53.9	60.2	45.4	41.7	46.0	31.5	32.7	44.1
Chen et al. [37] (*T* = 9)	-	-	-	-	-	-	-	-	-	-	-	-	-	-	-	46.3
Lie et al. [8] (*T* = 31)	40.8	46.0	41.3	57.1	47.0	52.8	39.9	42.3	55.2	72.4	44.7	53.3	47.7	33.3	34.9	47.3
HDPose [38] (*T* = 243)	37.8	40.7	37.7	39.6	42.4	50.2	39.8	40.2	51.8	55.8	42.2	39.8	41.0	27.9	28.1	41.0
DASTFormer [39] (*T* = 243)	36.8	39.7	39.3	34.3	40.9	50.6	36.8	36.7	50.9	59.0	41.4	38.4	37.9	25.3	25.8	**39.6**
STUNet [40] (*T* = 27)	43.5	44.8	43.9	44.1	47.7	56.5	44.0	44.2	55.8	67.9	47.3	46.5	45.7	33.4	33.6	46.6
Ours (*T* = 1)	36.3	42.8	40.2	57.5	44.7	48.2	37.2	38.9	53.9	74.1	42.7	55.0	43.7	32.7	34.1	45.7
**Protocol #2**	**Dir.**	**Disc.**	**Eat**	**Greet**	**Phone**	**Photo**	**Pose**	**Purch.**	**Sit**	**SitD.**	**Smoke**	**Wait**	**WalkD**	**Walk**	**WalkT**	**Avg.**
CEE-Net [19] (*T* = 1)	-	-	-	-	-	-	-	-	-	-	-	-	-	-	-	36.8
Zou et al. [29] (*T* = 1)	35.7	38.6	36.3	40.5	39.2	44.5	37.0	35.4	46.4	51.2	40.5	35.6	41.7	30.7	33.9	39.1
HTNet [33] (*T* = 1)	-	-	-	-	-	-	-	-	-	-	-	-	-	-	-	38.6
MHFormer [35] (*T* = 351)	31.5	34.9	32.8	33.6	35.3	39.6	32.0	32.2	43.5	48.7	36.4	32.6	34.3	23.9	25.1	34.4
PoseFormer [36] (*T* = 81)	32.5	34.8	32.6	34.6	35.3	39.5	32.1	32.0	42.8	48.5	34.8	32.4	35.3	24.5	26.0	34.6
Chen et al. [37] (*T* = 243)	32.6	35.1	32.8	35.4	36.3	40.4	32.4	32.3	42.7	49.0	36.8	32.4	36.0	24.9	26.5	35.0
Lie et al. [8] (*T* = 31)	31.6	35.1	34.2	38.6	37.3	38.9	31.3	32.9	45.2	51.1	36.3	37.0	35.8	24.8	26.9	35.8
HDPose [38] (*T* = 243)	31.0	33.2	30.6	31.9	33.2	39.2	31.1	30.7	42.5	45.0	34.1	30.7	32.5	22.0	23.0	**32.8**
DASTFormer [39] (*T* = 243)	31.1	33.7	33.8	29.4	34.0	39.6	30.3	31.4	43.5	49.7	36.0	31.3	32.8	22.0	22.6	33.4
STUNet [40] (*T* = 27)	34.3	35.7	34.9	36.6	37.5	42.7	33.1	36.0	44.4	53.7	38.5	33.5	38.4	26.0	28.4	36.9
Ours (*T* = 1)	31.0	34.8	34.8	39.9	37.6	38.8	31.1	32.0	46.4	52.9	37.1	38.3	36.2	27.0	29.8	36.6

**Table 7:** Comparison of the model size and GFLOPs.

Model	Model Size (MB)	GFLOPs	MPJPE (mm)
Chen et al. [37] (*T* = 9)	531 (CPN) + 903	NA	46.3
HTNet [33] (*T* = 27)	531 (CPN) + 11.6	NA	46.1
MHFormer [35] (*T* = 351)	531 (CPN) + 120	NA	43.0
Ours	405.9 (Real-Net) + 528.1 (Virtual-Net) + 590.6 (FM, MLP + DenseFC)	18.17	45.7
Ours	405.9 (Real-Net) + 528.1 (Virtual-Net) + 1.5 (FM, GCN + GCN)	18.13	46.4

## Data Availability

Data are contained within the article.
